# Supplementary material for: Examining protective effects of SARS-CoV-2 neutralizing antibodies after vaccination or monoclonal antibody administration
Source: Nat Commun. 2023 Jun 17;14:3605. doi: 10.1038/s41467-023-39292-w (PMC10276829; doi:10.1038/s41467-023-39292-w)
Supplement: Supplementary file 1 — Supplementary Information [file 41467_2023_39292_MOESM1_ESM.pdf]

Supplementary Materials for

Examining Protective effects of SARS-CoV-2 Neutralizing antibodies after vaccination or monoclonal antibody administration

Dean Follmann, PhD[1*]; Meagan P. O'Brien, MD[2], Jonathan Fintzi, PhD[1], Michael P. Fay, PhD[1], David Montefiori, PhD[3], Allyson Mateja, MSPH[4], Gary A. Herman, MD[2], Andrea T. Hooper, PhD[2], Kenneth C Turner, PhD[2], Kuo- Chen Chan, PhD, MS[2], Eduardo Forleo-Neto, MD, MSc[2], Flonza Isa, MD[2], Lindsey R Baden, MD[5], Hana M El Sahly,MD[6], Holly Janes, PhD[7], Nicole Doria-Rose, PhD[8], Jacqueline Miller, MD[9], Honghong Zhou, PhD, MS[9], Weiping Dang, PhD[9], David Benkeser, PhD, MPH[10], Youyi Fong, PhD[7,11,12], Peter B. Gilbert, PhD[7,11,12], Mary Marovich, MD[1], and Myron S. Cohen, MD[13]

Affiliations

[1]Biostatistics Research Branch, National Institute of Allergy and Infectious Diseases, National Institutes of Health, Bethesda, MD, USA.

[2]Regeneron Pharmaceuticals, Inc., Tarrytown, NY, USA.

[3]Department of Surgery, Duke University Medical Center; Durham, NC, USA.

[4]Clinical Monitoring Research Program Directorate, Frederick National Laboratory for Cancer Research, Frederick, MD, USA.

[5]Brigham and Women's Hospital, Boston, MA, USA.

[6]Department of Molecular Virology and Microbiology, Baylor College of Medicine, Houston, TX, USA.

[7]Vaccine and Infectious Disease Division, Fred Hutchinson Cancer Research Center, Seattle, WA, USA.

[8]Vaccine Research Center, National Institutes of Health, Bethesda, MD, USA.

[9]Moderna, Inc., Cambridge, MA, USA.

[10]Department of Biostatistics and Bioinformatics, Rollins School of Public Health, Emory University, Atlanta, GA, USA.

[11]Public Health Sciences Division, Fred Hutchinson Cancer Research Center, Seattle, WA, USA.

[12]Department of Biostatistics, University of Washington, Seattle, WA, USA.

[13] Institute for Global Health and Infectious Diseases. The University of North Carolina at Chapel Hill, NC, USA.

Correspondence to: dfollmann@niaid.nih.gov

Materials and Methods

Section 1: Deconstruction analysis

We deconstruct the total effect of vaccination (V) at a neutralization titer of 1000 IU50/ml into a part (A) due to extant circulating and possibly mucosal antibodies and a part (O) due to all other aspects of vaccine induced immunity including memory B-cells, the anamnestic response to exposure, T-cells etc. The extant circulating and possibly mucosal antibodies are quantified by neutralization titer but include non-neutralizing functions such as ADCP and ADCC. We approximate part A by the effect of monoclonal antibodies at a titer of 1000 IU50/ml. We assume the proportional hazards models for the vaccine and mAb trials are correctly specified, that there are no unmeasured confounders, and that the individuals at risk at the time of exposure are exchangeable. At 1000 IU50/ml, the hazard ratios for vaccine vs placebo and mAb versus placebo are 0.03 = (1-0.97) and 0.08 = (1-0.92), respectively.

We decompose the total vaccine effect as measured by the hazard ratio as

$$V = A \times O. \qquad (S1)$$

$$0.03 = 0.03^{P} \times 0.03^{1-P} \qquad (S2)$$

$$0.03 = 0.08 \times 0.03^{1-P} \qquad (S3)$$

We estimate P = log(0.08)/log(0.03) = 0.72, thus 72% is the proportion of the total vaccine effect due to extant circulating and mucosal antibodies.

Section 2: Probability vaccine induced protection is due to antibody (PA)

Our development is based on the *causes of effects* approach to causal modeling. We begin by considering a simple three arm randomized trial. Let Y(0), Y(1),Y(2) be the potential outcomes (COVID-19 indicators) for an individual randomized to Z=0,1,2 or placebo, mAb, vaccine, and let Y(Z) be the actual outcome for an individual assigned to arm Z, with Y=1 denoting an event during the study, and Y=0 for no event. We invoke a monotonicity assumption that Y(0)≥ Y(1) ≥ Y(2), i.e., individuals infected on a

more substantial regimen remain infected on a less substantial regimen, and individuals infected on a less substantial regimen may be uninfected on a more substantial regimen. Here we order the regimens as placebo is equal to or less substantial than mAb which is equal to or less substantial than vaccine. As a numerical illustration, suppose that a large 1:1:1 randomized trial is conducted with counts N_0, N_1, N_2 = 100, 20, 5 where N_Z is the number of COVID-19 cases on arm Z (Table S3). Thus, we estimate PE = 1-20/100=0.80 and VE=1-5/100=0.95. Under the monotonicity assumption, we impute counts for the three sets of potential outcomes in the next to last column.

Table S3 explained

A protected person is one who would be infected on placebo but not infected on vaccine. We thus estimate that 95 individuals were protected by the vaccine (due to either extant antibodies or other agents [e.g., antibodies from B-cells or T-cells]). Since 80 were protected in the mAb arm, we estimate the probability protection is due to antibody is 80/95.

More formally, the probability vaccine induced protection is due to antibody PA is expressed as

P(Y(1)=0 | Y(0),Y(1),Y(2) = 1,0,0 or 1,1,0) = PE/VE. This result follows from the following argument.

$$P(Y(1) = 0 \mid \{Y(0), Y(1), Y(2)\} = \{1,0,0\} \ or \ \{1,1,0\}) \quad \text{(S4)}$$

$$= \frac{P(\{Y(0), Y(1), Y(2)\} = \{1,0,0\})}{P(\{Y(0), Y(1), Y(2)\} = \{1,0,0\} \ or \ \{1,1,0\})} \quad (S5)$$

$$= \frac{P(\{Y(1),Y(2)\}=\{0,0\} \mid Y(0)=1) \, P(Y(0)=1)}{P\big(\{Y(1), Y(2)\} = \{0,0\} \, or \, \{1,0\} \,\big|\, Y(0) = 1\big) P(Y(0)=1)} \quad (S6)$$

$$= \frac{P(Y(1) = 0 \mid Y(0) = 1) \, P(Y(0) = 1)}{P(Y(2) = 0 | Y(0) = 1) P(Y(0) = 1)} \quad (S7)$$

$$= \frac{PE\ P(Y(0) = 1)}{VE\ P(Y(0) = 1)} \qquad (S8)$$

$$= \frac{PE}{VE} \qquad (S9)$$

Equations (S5) and (S6) follow from the definition of conditional probability, the numerator of (S7) follows from monotonicity, and (S8) follows from the definitions of PE and VE, i.e., that protection was caused by the mAb and by the vaccine, respectively.

While the above development was for a 1:1:1 randomized three-arm trial and a simple analysis with VE and PE defined as 1 minus the ratio of counts, it applies to the proportional hazards estimates of PE and VE from separate trials under assumptions. To see this, suppose that at a given level of antibody, e.g., *Ab* IU50/ml, $X^v(0)$, $X^v(1)$, $X^v(2)$ represent the potential outcomes for an individual in the next instant of time in the mRNA-1273 vaccine trial and $X^m(0)$, $X^m(1)$, $X^m(2)$ is analogously defined for the COV-2969 mAb trial. Thus, we allow that the placebo attack rates in the two trials to be different. Assume the following: monotonicity, that the proportional hazards models for VE(*Ab*) and PE(*Ab*) are correctly specified, that the VE(*Ab*) and PE(*Ab*) functions are the same for both trials, and represent the causal effects of vaccination and assignment to an antibody titer of *Ab* IU50/ml and the causal effect of assignment to mAb antibody titer of *Ab* IU50/ml. Let t = v, m index the vaccine and mAb trial respectively. By replacing Y(0),Y(1),Y(2) in the above argument with $X^t(0)$, $X^t(1)$, $X^t(2)$ we have that the probability an individual with antibody level *Ab* IU50/ml would be protected in the next instant of time on the mAb arm is PE(*Ab*)/VE(*Ab*). Thus, under these assumptions the probability of mAb protection given vaccine protection for an individual with titer *Ab* applies to either trial and the PE(*Ab*) and VE*(Ab*) curves can be estimated using the mAb and vaccine trial, respectively, and do not require a three-arm trial.

Section 3: Definitions of COVID-19 disease

COV-2069 mAb Prevention Trial COVID-19 Disease Definition

Participants had to be asymptomatic, PCR negative, and seronegative at baseline. Then if they developed symptoms consistent with COVID-19 (any of those listed below) and nasopharyngeal PCR confirmed SARS-CoV-2, they had confirmed COVID-19 disease.

1. Fever ≥38 degrees C or feverish
2. Sore Throat
3. Cough
4. Shortness of breath/difficulty breathing (nasal flaring*)
5. Chills
6. Nausea
7. Vomiting
8. Diarrhea
9. Headache
10. Red or watery eyes (conjunctivitis)
11. Body aches such as muscle pain or joint pain (myalgia, arthralgia)
12. Loss of taste/smell
13. Fatigue (fatigue or general malaise or lethargy*)
14. Loss of appetite or poor eating/feeding
15. Confusion
16. Dizziness
17. Pressure/tightness in chest
18. Chest pain

19. Stomachache (abdominal pain*)

20. Rash

21. Sneezing

22. Runny nose

23. Sputum/phlegm

Other

*Signs and symptoms observed in pediatric subjects

COVE Trial COVID-19 Disease Definition

COVID-19 disease was defined as symptomatic disease (based on the criteria below) AND the participant had at least 1 nasopharyngeal swab or saliva sample (or respiratory sample, if hospitalized) positive for SARS-CoV-2 by RT-PCR.

- The participant must have experienced at least TWO of the following systemic symptoms: fever (≥38 degrees C), chills, myalgia, headache, sore throat, new olfactory and taste disorder(s), OR
- The participant must have experienced at least ONE of the following respiratory signs/symptoms: cough, shortness of breath or difficulty breathing, OR clinical or radiographical evidence of pneumonia.

Section 4: Relationship between Duke and VRC pseudo-virus neutralization assays

Sixty-eight paired samples were assayed on both the Duke and VRC pseudo-virus neutralization assay. Figure S6 displays a 45-degree line (red) and a fitted line using standard Deming regression (dashed

blue) which assumes the ratio of the error variances for the two assays are equal. The intercept and slope (standard errors) from the Deming regression are -0.229 (0.109) and 1.056 (0.041).

Section 5: Conversion of reciprocal dilutions to IU50/ml

Reciprocal dilution titers for ID50 from the Duke assay were converted to International Units by the formula IU50/ml = ID50 x 0.242 where ID50 is the reciprocal dilution titer for the 50% inhibitory dilution (ID50). Thus, a titer of 1000 IU ID50/ml corresponds to a reciprocal dilution ID50 titer of 4132.24 because 4132.24 x 0.242 = 1000.00. Details are provided in Gilbert et al.(*8*)

Section 6: Modelling antibody kinetics of mRNA-1273

We modeled the kinetics of mRNA1273-elicited neutralizing antibodies using data from 34 participants separate from the COVE trial, measured at days 57, 119, and 209 following the first dose of vaccine. Figure S5, Doria-Rose et al.(*39*) Antibody over time was characterized using a hierarchical Bayesian exponential decay model, adjusting for study day, age, and sex as "fixed effects". The model accounts for within-subject autocorrelation via random intercepts. The functional form of the mean model reflects the assumption that the rate at which antibodies decay is proportional to their abundance. We also integrated out missing values for three measurements that were below the assay limit of detection (LoD). Priors were assigned so that they were weakly informative on the scale of the data. A detailed specification of the model and model-fitting procedure are given below. After adjusting for age and sex, we estimate that neutralizing antibodies decay at a rate of $\widehat{\beta_1}$ = -0.0043 log10 titer units (95% CI: -0.0049, -0.0037) per day. This corresponds to an estimated half-life of log(2)/{log(10) x 0.0043} = 70 days (95% CI: 62, 81), which is in general agreement with the half-life estimate of 69 days (95% CI: 61, 76) reported in Doria-Rose, et al.(*39*)

The model for log10 nAb titer for the ith individual at time t, Y_{it} is formulated as follows

$$Y_{it} \sim N(\mu_{it}, \sigma^2), \tag{S10}$$

$$\mu_{it} = \beta_0 + \beta_1 t + \beta_2 \mathrm{Age}_i + \beta_3 \mathrm{Male}_i + b_i \tag{S11}$$

$$\beta_0 \sim \mathrm{Student} - t(\mathrm{d.f.} = 3, \mathrm{location} = 2.2, \mathrm{scale} = 2.5^2), \tag{S12}$$

$$\beta_1,\ \beta_2,\ \beta_3 \sim \mathrm{Normal}(0, 2.5^2), \tag{S13}$$

$$b_i \sim \mathrm{Normal}(0, \tau^2), \tag{S14}$$

$$\sigma,\ \tau \sim \mathrm{Exponential}(1). \tag{S15}$$

The Student-t prior for β_0 is parameterized by its degrees of freedom, location, and scale. Note that observations above the assay LoD make Gaussian density contributions to the likelihood. To account for censoring due to limits of detection, we integrate out the missing observations. Hence, each censored observation contributes a Gaussian CDF term to the likelihood. Hence, the likelihood is

$$\mathrm{L}(\mathbf{Y} \mid \theta) = \prod_i \prod_t [(1 - \delta_{it})\phi(Y_{it}; \mu_{it}, \sigma^2) + \delta_{it}\Phi(\mathrm{LoD}; \mu_{it}, \sigma^2)], \tag{S16}$$

where $\phi(\mu_{it},\ \sigma^2)$ and $\Phi(\mu_{it},\ \sigma^2)$ are Gaussian probability density and cumulative density functions, respectively, and δ_{it} denotes either below ($\delta_{it} = 1$) or above ($\delta_{it} = 0$) the LoD.

To fit the model, we ran four MCMC chains for 2,000 iterations each, discarding the first 1,000 iterations of each chain as warmup, and combining the remaining samples from all chains. Convergence of MCMC was assessed visually and by verifying that all potential scale reduction factors were less than 1. We also separately assessed the sensitivity of our inference about fixed effects to alternative prior specifications, using more diffuse priors for the "fixed effect" parameters and a student-t error distribution, and found that the posterior mean point estimate of the rate of decay (which is the target of inference for use in the second-stage time to event model) was essentially unchanged.

Section 7: Monoclonal antibody risk model

The risk of acquiring COVID-19, on day d post injection, as a function of that day's imputed log10 neutralization titer was specified by a 3-parameter logistic curve

$$h(d) = h_0(d)\{\ (1 - Z) + Z[\theta + (1 - \theta)\ expit\{\ \beta_0 + \beta_1\ Ab(d)\ \}\ ]\ \} \tag{S17}$$

where Ab(d) is an individual's predicted log10 ID50 neutralization titer on day d post injection, expit(a)=exp(a)/{1+exp(a)}, Z=1 for mAb recipients and 0 for placebo recipients, and $h_0(d)$ is the risk of COVID-19 on day d for a placebo recipient. Protective efficacy as a function of circulating antibody, Ab is given by

$$PE(Ab\ )\ =\ 1 - \{\ \theta +\ (1 - \theta)\ expit(\beta_0 + \beta_1\ Ab)\ \}. \tag{S18}$$

This curve reflects anticipated effects of antibody abundance on the risk of COVID-19. Suppose that $\beta_1\ < 0$. As Ab goes to infinity, PE(Ab) approaches 1 - $\theta \leq 1$, the maximal protective efficacy. As Ab goes to minus infinity (i.e., ID50 goes to 0), PE(Ab) approaches 0 so that with no antibody in either arm, the risk of COVID-19 is the same. The ratio $-\beta_0/\beta_1$ determines the level of Ab where the maximal protective efficacy of $(1 - \theta)$ is halved. This model was estimated using a Poisson approximation with a term for the time since injection. As a sensitivity analysis we fit a log-linear model for the risk of COVID-19 using Cox regression with hazard function

$$h(d) = h_0(t)\ \exp\{\ Z[\ \beta_0 + \beta_1\ Ab(d)]\}. \tag{S19}$$

We obtain 95% pointwise confidence intervals (CIs) for the parameters in (S17) via the bootstrap and propagate uncertainty about the relationship between concentration and titer by a random draw from the bivariate distribution of the slope and intercept as estimated from the 18 paired samples. Details follow. We first estimated the parameters from the below equation

$$Y_i = \eta_0, +\ \eta_1 X_i\ \ + e_i \tag{S20}$$

using Y_i , X_i i=1,…18 the paired samples of log10 (ID50), log10 concentration of antibody. Estimation was done by ordinary least squares. Denote the estimated parameters as $\widehat{\eta_0}, \widehat{\eta_1}$ and the estimated covariance matrix of $\widehat{\eta_0}, \widehat{\eta_1}$ by $\hat{C}$.

For a single bootstrap sample of the 1630 individuals in the COV-2069 trial we first sampled $\eta_0^b,\ \eta_1^b$ from a bivariate normal distribution with mean $\widehat{\eta_0}, \widehat{\eta_1}$ and covariance $\hat{C}$. From this $\eta_0^b,\ \eta_1^b$ we generated individualized log10(ID50) decay curves by creating, for each day and each person the predicted log10 ID50 titer, y_{jd}, according to the equation

$$y_{jd} = \eta_0^b, + \eta_1^b \ , x_{jd} \qquad \text{(S21)}$$

where x_{id} was the antibody concentration for person i=1,…1630 on day d=1,…,240 post injection. We then sampled the 1630 participants in the analysis set with replacement. Using these 1630 sampled participants we estimated the parameters in the hazard function (MA). We did this 10,000 times resulting in 10,000 estimates of $\theta,\ \beta_0, \beta_1$. We calculated percentile bootstrap confidence intervals for different functions of $\theta,\ \beta_0, \beta_1$ by determining the .025 and .975 percentiles of the 10,000 estimates. For example $\widehat{\mathrm{PE}^{\mathrm{b}}}(\mathrm{Ab})$, b=1 ,…,10,000 where $\widehat{\mathrm{PE}^{\mathrm{b}}}(\mathrm{Ab})$ is equation (S8) with the parameters replaced by their estimated values using the bth bootstrapped data set.

<u>Section 8: Vaccine induced antibody risk model</u>

The risk of acquiring COVID-19, on any day t, as a function of the neutralization titer on that day, was estimated using a standard Cox proportional hazards regression model similar to that of Gilbert et al.,(*8*) but with time varying antibody and calendar time as the operational timescale. The model adjusts for X_1 =Minority Status, X_2 = High Risk stratum, and X_3 = Risk Score resulting in the hazard function

$$h(t) = h_0(t) \exp \{ X_1\alpha_1 + X_2\alpha_2 + X_3\alpha_3 + \ Z[\ \beta_0 + \beta_1\, Ab(d(t))]\ \} \qquad \text{(S22)}$$

where t is the number of days since 1 July 2020, d(t) is the number of days post day 57 after 1st dose on calendar day t, Z is 1 for those in the vaccine arm and 0 otherwise, Ab(d) is the imputed log10 antibody titer on day d post day 57 after 1st dose, and Ab(d) = Ab(0) – 0.0043 d.

Vaccine efficacy as a function of circulating antibody titer Ab is specified as

$$VE(Ab) = 1 - exp(\beta_0 + \beta_1 Ab) \qquad \text{(S23)}$$

Instead of assessing Day 57 antibody in all 14,202 vaccine arm participants, Gilbert et al., (8) used a case-cohort design comprised of a stratified random sample of 1010 participants who comprised the immunogenicity subcohort, plus 36 disease cases (5 of which were in the set of 1010). Details of the case-cohort sampling design for the immune correlates of risk sub-study and derivation of inverse probability of sampling weights (IPSW) are described in Gilbert, et al.(*8*) Here, we reanalyze data used in the day 57 correlates analysis. We estimate controlled VE using covariate adjusted inverse probability of sampling weighted Cox proportional hazards models. Study participants are weighted by their inverse probability of being sampled in the immunogenicity subcohort, as described in Gilbert, et al.,(*8*) so that the weighted subcohort matches the study cohort on demographic strata. Let π_i denote each participant's sampling weight, which for a non-case in risk-demographic-treatment stratum k, is equal to the ratio of numbers of participants in the phase 1 and phase 2 participants in that stratum, N_k/n_k. Each case receives weight equal to the ratio of the number of cases in the phase 1 and phase 2 datasets in their respective treatment arm, N_z/n_z.

We obtain 95% pointwise confidence intervals via a two-step procedure similar to COV-2069. For each bootstrap sample we sample the COVE participants with replacement. We propagate uncertainty about the rate of neutralizing antibody decay estimated from the previously described antibody model by using a random draw from the posterior distribution of the rate of decay in each bootstrap iteration to predict neutralization titers at each day post day 57. Following Gilbert, et al.,(*8*) the resampling step of COVE participants was stratified by groupings of risk demographic strata and randomization to the immunogenicity subcohort.

Section 9: Model goodness-of-fit

To assess goodness-of-fit we compared the Kaplan-Meier curves of cumulative incidence with the model-based cumulative incidence, see below for the COVE and COV-2069 studies, respectively. A feature of these data is that the placebo arm is fit quite well because the vast majority of cases are from the placebo arms in both trials. We see that the model based cumulative incidence curve for the vaccine arm lies within the confidence band for the Kaplan-Meier curve and nearly so for the mAb arm which is more variable due to having few events in that arm (See Figure S7).

Another approach to assess goodness-of-fit for proportional hazards models is to see if the regression parameters (slopes) differ for early versus late follow-up. We thus interacted the coefficients for arm and arm*log10(titer) for COVE with an indicator of early versus late follow-up. Neither interaction term was significant for COVE. For COV-2069 the model could not be fit due to too few events.

Figure S1: Cumulative incidence of COVID-19 by study day for the two trials. Panel (A) is the COV-2069 prevention trial with the mAb combination casirivimab + imdevimab (CAS+IMD), Panel B is the COVE vaccine trial. Cases prior to day 8 (mAb) and day 63 (vaccine) are not included. The p-value is based on a two-sided log-rank test.

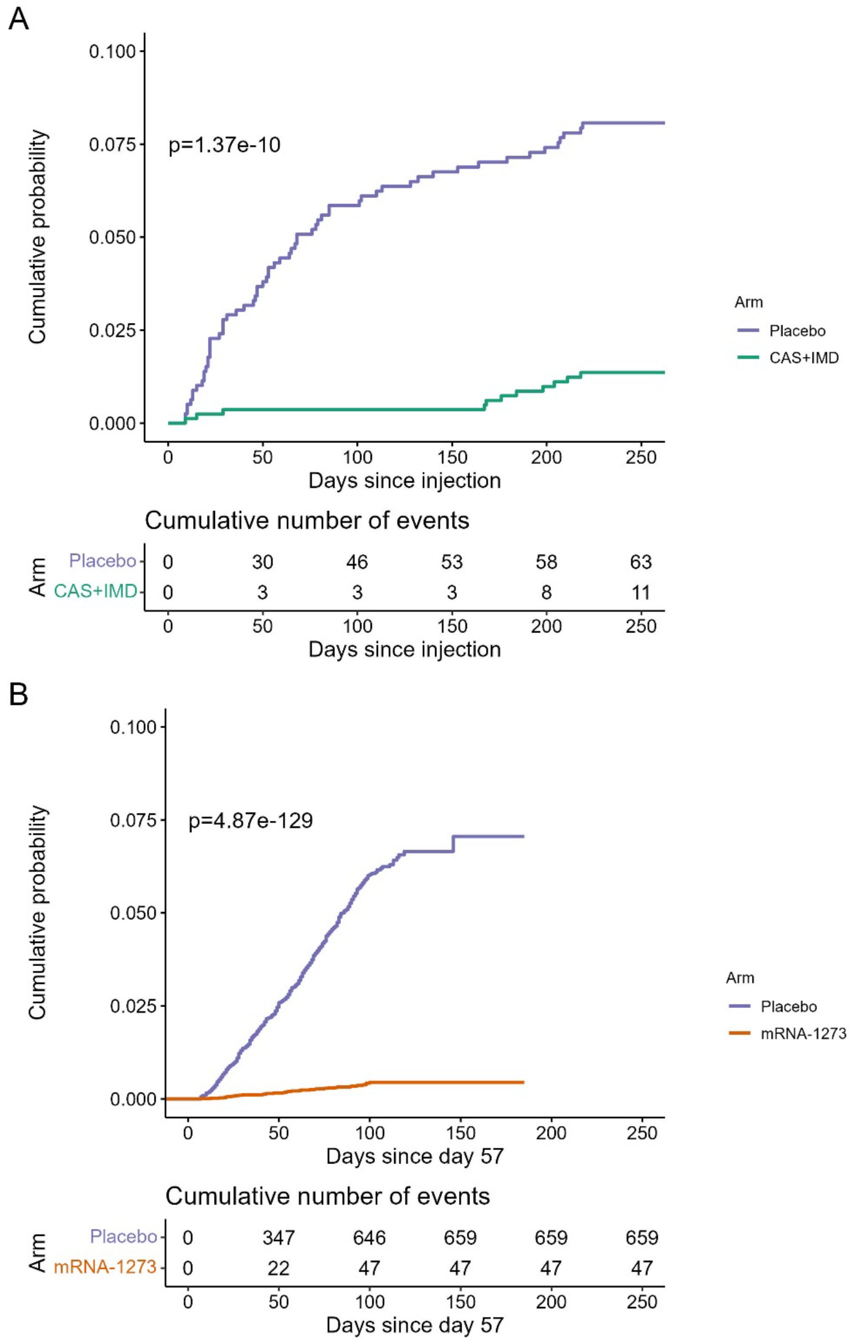

Figure S2: The protective efficacy (PE) of casirivimab and imdevimab against RT-qPCR-confirmed SARS-CoV-2 infection regardless of symptoms. Shaded area corresponds to 95% bootstrap pointwise confidence intervals while the solid green curve is the estimated PE. Infection counts were 32 in the mAb arm and 100 in the placebo arm. Dots denote the predicted neutralization titer at the time of infection whether actual (mAb arm) or counterfactual/hypothetical (placebo arm) because predicted neutralization titer depends only on sex, weight, and time since injection.

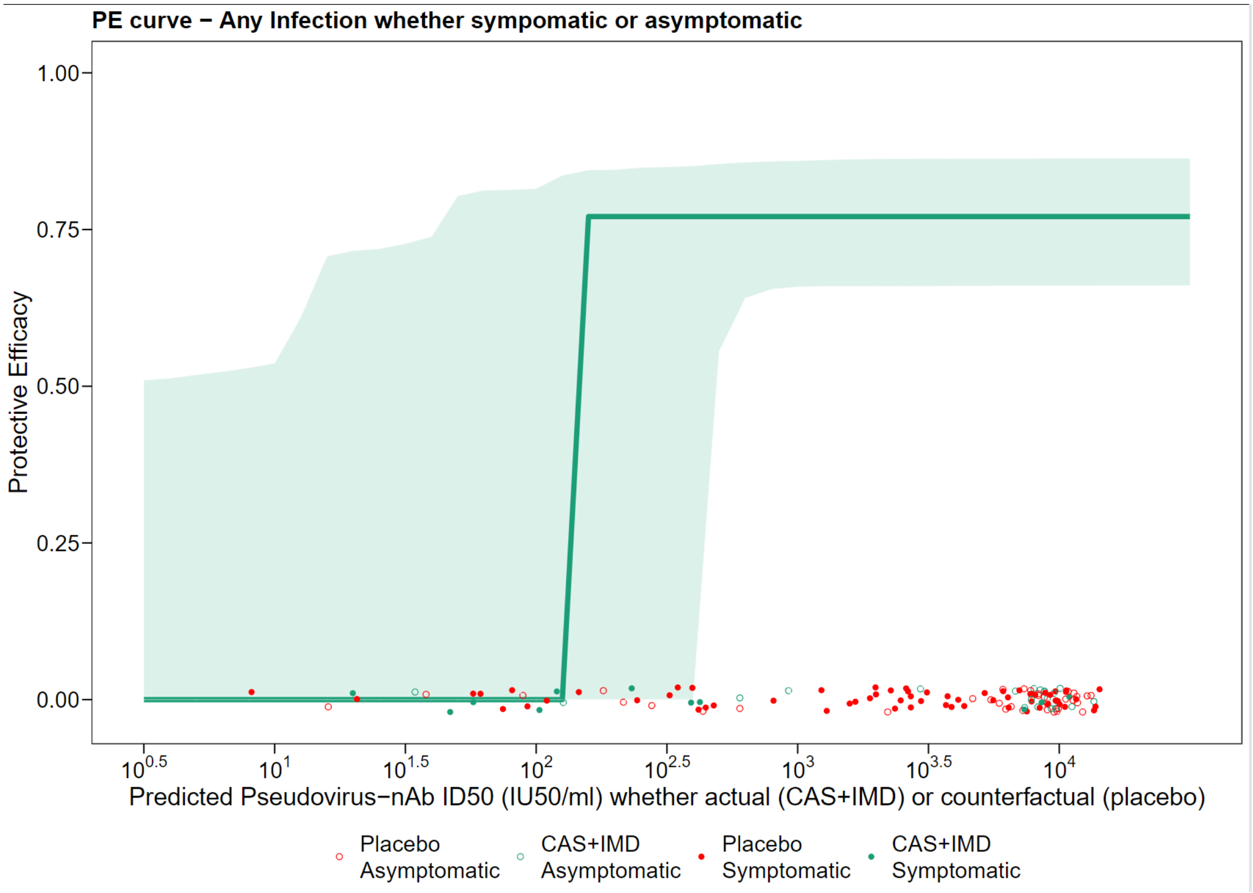

Figure S3: The protective efficacy (PE) of casirivimab and imdevimab against RT-qPCR-confirmed SARS-CoV-2 asymptomatic infection. Asymptomatic infection was assessed by RT-qPCR weekly through the 1st month of follow-up and then participant driven testing, e.g., screening for school, work or close contact exposure. Shaded area corresponds to 95% bootstrap pointwise confidence intervals while the solid green curve is the estimated PE. Infection counts were 21 in the mAb arm and 37 in the placebo arm. Dots denote the predicted neutralization titer at the time of infection whether actual (mAb arm) or counterfactual/hypothetical (placebo arm) because predicted neutralization titer depends only on sex, weight, and time since injection.

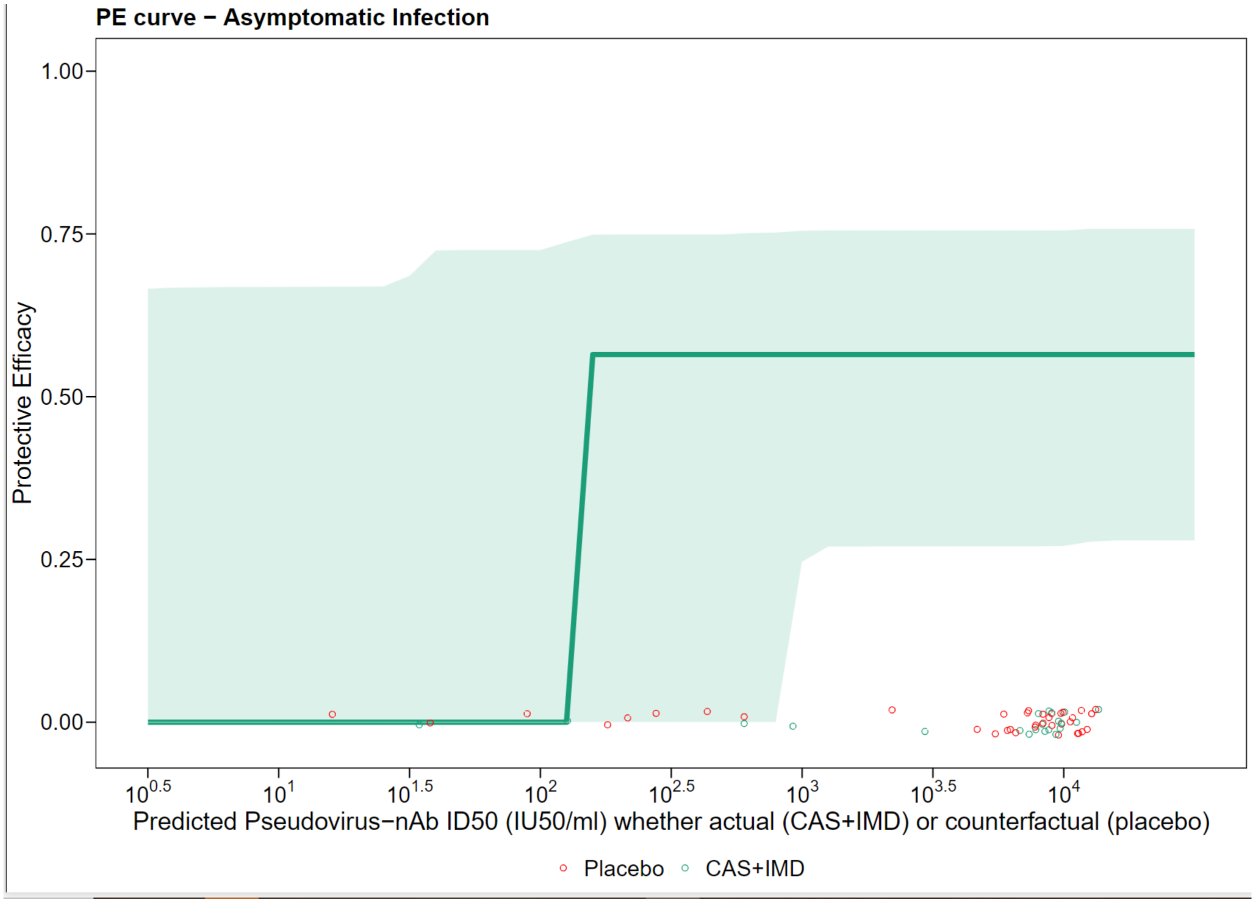

Figure S4: Protective Efficacy (PE) of casirivimab + imdevimab mAbs (solid green curve) and Vaccine Efficacy (VE) of mRNA-1273 (dashed orange curve) against COVID-19 as a function of predicted pseudo-virus neutralization titer at the time of exposure. Shaded area provides 95% pointwise confidence intervals. PE and VE curves cover the distribution of titers achieved during follow-up with no extrapolation. Both PE and VE curves are based on a log-linear function of predicted neutralization titer and estimated using Cox regression.

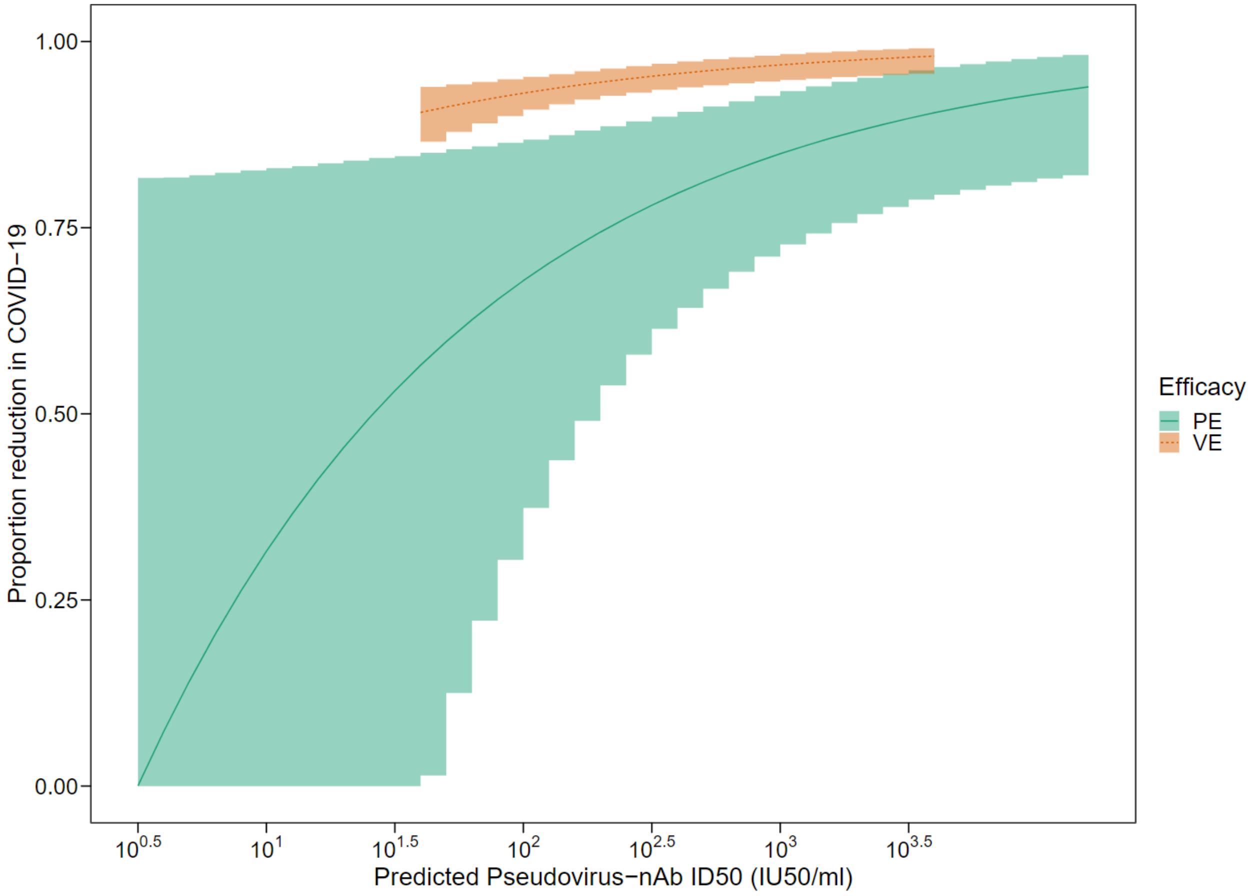

Figure S5: Pseudo-virus neutralization titers from the VRC at 3 time points by age and sex. Data reported in Doria-Rose et al.(*39*)

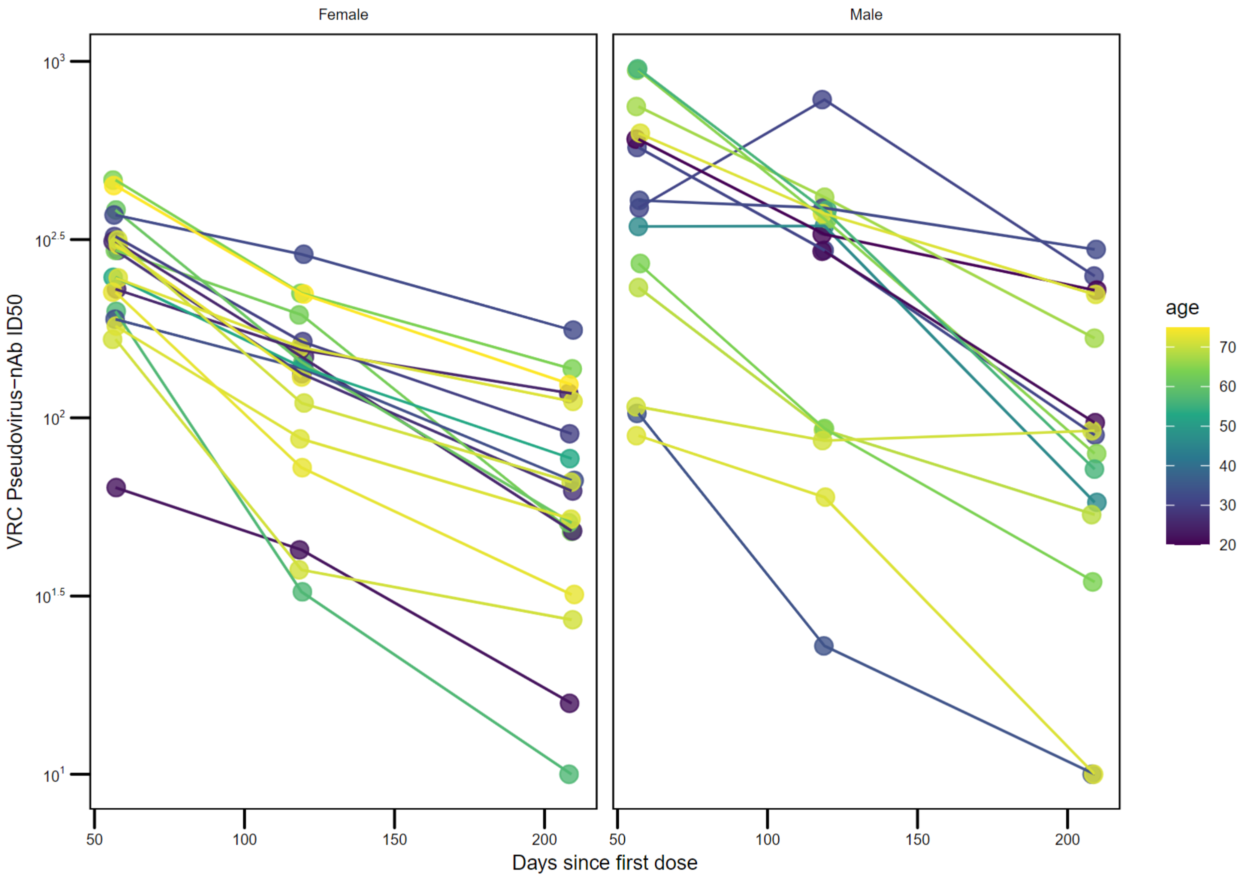

Figure S6: A scatterplot of 68 paired samples assayed using the Duke and VRC pseudo-virus neutralization assay. Solid red and dashed blue lines denote a 45-degree line through the origin and from standard Deming regression, respectively.

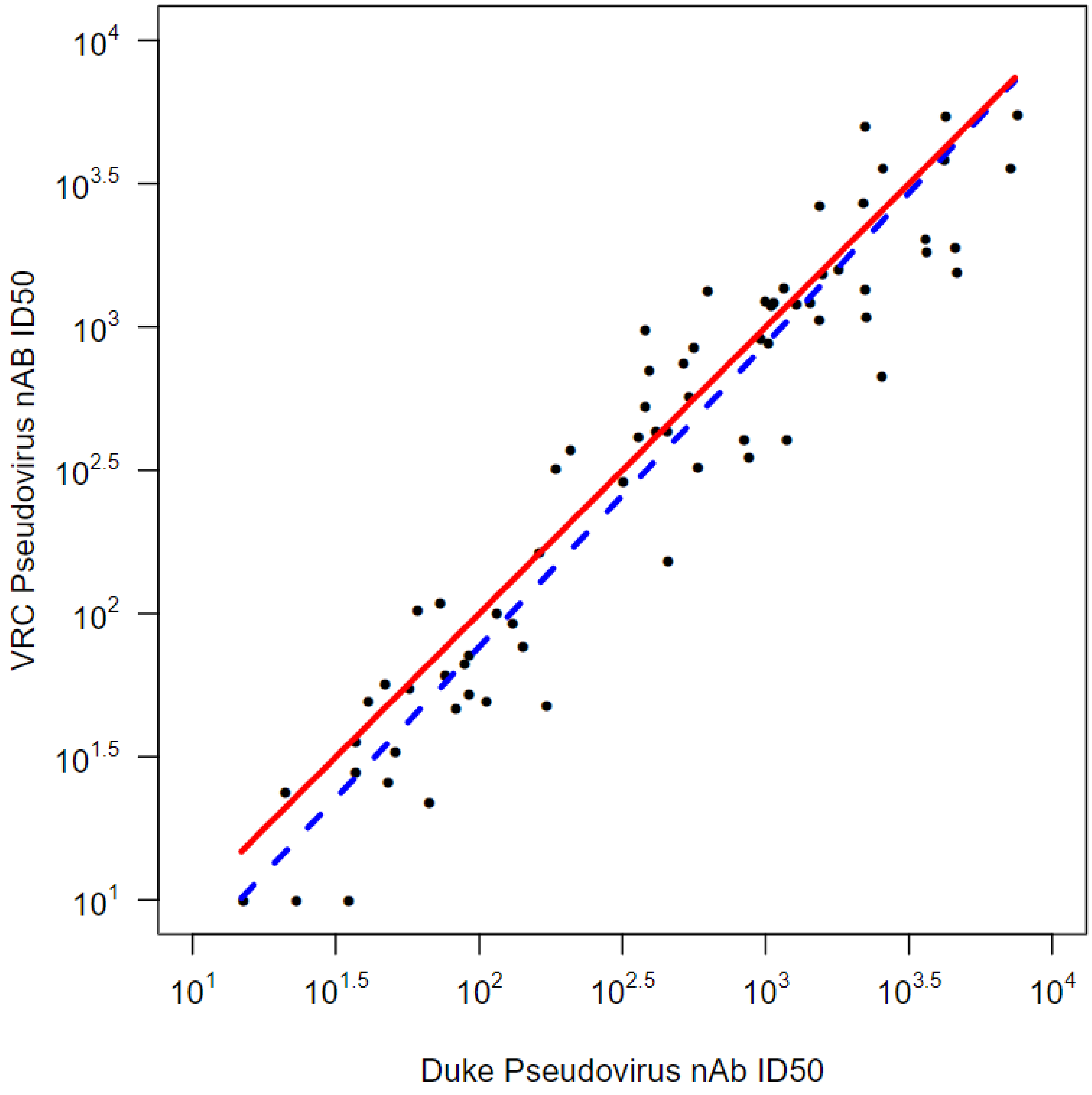

Figure S7: Model Goodness-of-Fit Testing. The solid black curves denote the Kaplan-Meier estimates while the red dashed curves denote the model-based estimates.

A.COVE VACCINE TRIAL

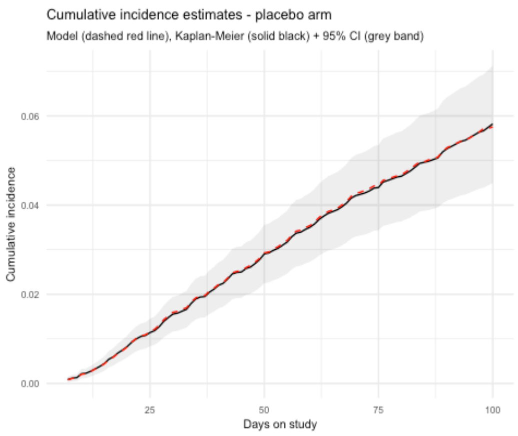

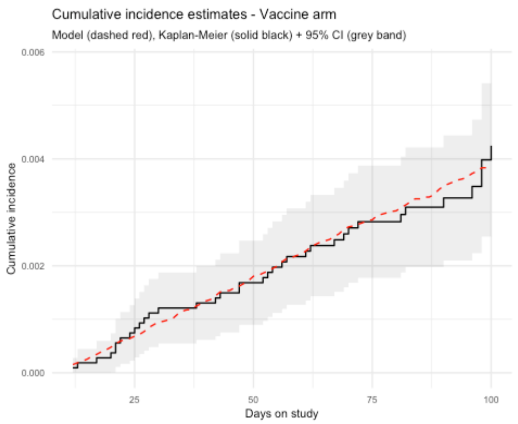

B. COV-2069 MONOCLONAL ANTIBODY TRIAL

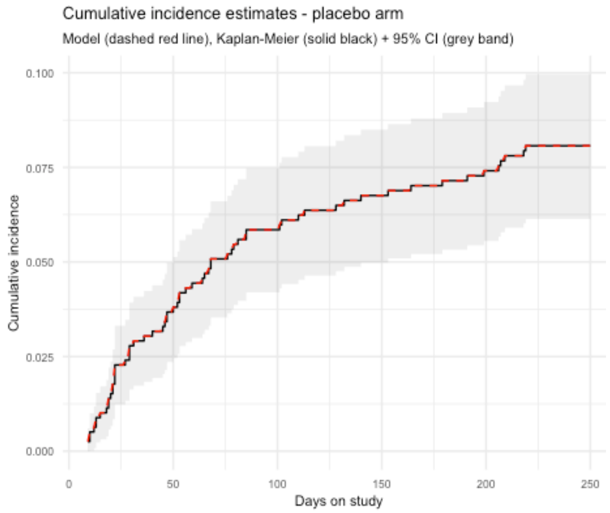

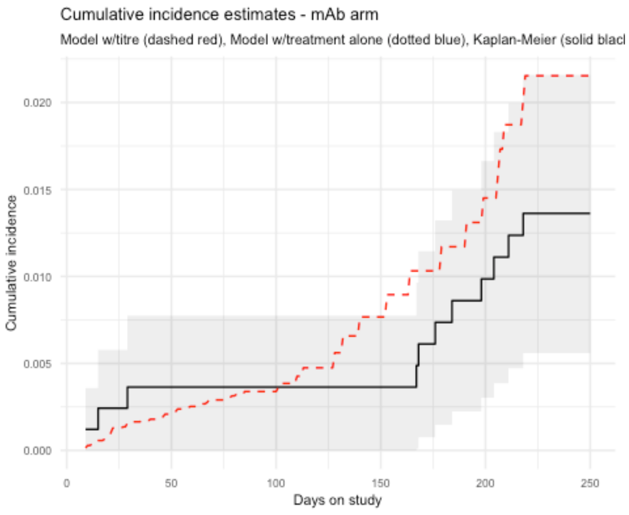

Table S1: Disposition and baseline characteristics of COV-2069 participants with no evidence of infection at baseline.

	Overall	Casirivimab and imdevimab arm	Placebo arm
N Randomized	1682	840	842
N Infected Week 1	51 (3.0)	10 (1.2)	41 (4.9)
N Dropped Week 1	1 (0.1)	1 (0.1)	0 (0.0)
N in Analysis Set	1630	829	801
Age, years			
Mean (SD)	42.34 (15.88)	42.55 (16.28)	42.12 (15.47)
>= 50, n (%)	596 (36.6)	311 (37.5)	285 (35.6)
Sex assigned at birth, n (%)			
Male	763 (46.8)	375 (45.2)	388 (48.4)
Female	867 (53.2)	454 (54.8)	413 (51.6)
Race, n (%)			
White	1396 (85.6)	721 (87.0)	675 (84.3)
Black or African American	152 (9.3)	70 (8.4)	82 (10.2)
Asian	46 (2.8)	24 (2.9)	22 (2.7)
American Indian or Alaska Native	7 (0.4)	2 (0.2)	5 (0.6)
Native Hawaiian or Pacific Islander	3 (0.2)	1 (0.1)	2 (0.2)
Other	26 (1.6)	11 (1.3)	15 (1.9)
Ethnicity, n (%)			
Hispanic or Latino	700 (42.9)	335 (40.4)	365 (45.6)
Not Hispanic or Latino	920 (56.4)	490 (59.1)	430 (53.7)
Other	10 (0.6)	4 (0.5)	6 (0.7)
Weight, mean kg (SD)	81.30 (19.47)	81.02 (19.12)	81.60 (19.84)

Table S1: Disposition and baseline characteristics of COV-2069 participants with no evidence of infection at baseline.

	**Overall**	**Casirivimab and imdevimab arm**	**Placebo arm**
Body-mass index, kg/m^2			
Mean (SD)	28.50 (6.15)	28.39 (5.94)	28.60 (6.37)
>= 30, n (%)	556 (34.3)	284 (34.4)	272 (34.1)
Risk of COVID-19 Acquisition			
Healthcare Worker or First Responder, n (%)	185 (11.3)	90 (10.9)	95 (11.9)
Mask Wearing in any indoor or outdoor, crowded or non-crowded place, n (%)			
Yes	750 (46.0)	383 (46.2)	367 (45.8)
No	22 (1.3)	9 (1.1)	13 (1.6)
Unknown	858 (52.6)	437 (52.7)	421 (52.6)
Do People Wear Masks in Home, n (%)	788 (48.9)	382 (46.5)	406 (51.3)
Household Size			
1	1072 (65.8)	535 (64.5)	537 (67.0)
2	376 (23.1)	203 (24.5)	173 (21.6)
3	105 (6.4)	51 (6.2)	54 (6.7)
4	52 (3.2)	27 (3.3)	25 (3.1)
>4	25 (1.5)	13 (1.6)	12 (1.5)

Week 1 defined as days 1-7

Table S2: Characteristics of the COVE analysis set by arm.

	Overall	mRNA-1273	Placebo
N Randomized	28281	14202	14079
N Infected Before Day 63	107 (0.4)	8 (0.1)	99 (0.7)
N Dropped Before Day 63	126 (0.4)	52 (0.4)	74 (0.5)
N in Analysis Set	28048	14142	13906
Age, years			
Mean (SD)	51.68 (15.51)	51.65 (15.45)	51.70 (15.56)
>= 50, n (%)	15876 (56.6)	8023 (56.7)	7853 (56.5)
Sex assigned at birth, n (%)			
Male	14644 (52.2)	7332 (51.8)	7312 (52.6)
Female	13404 (47.8)	6810 (48.2)	6594 (47.4)
Race, n (%)			
White	22419 (79.9)	11305 (79.9)	11114 (79.9)
Black or African American	2659 (9.5)	1356 (9.6)	1303 (9.4)
Asian	1310 (4.7)	624 (4.4)	686 (4.9)
American Indian or Alaska Native	216 (0.8)	107 (0.8)	109 (0.8)
Native Hawaiian or Pacific Islander	66 (0.2)	36 (0.3)	30 (0.2)
Other	1378 (4.9)	714 (5.0)	664 (4.8)
Ethnicity, n (%)			
Hispanic or Latino	5502 (19.6)	2785 (19.7)	2717 (19.5)
Not Hispanic or Latino	22288 (79.5)	11224 (79.4)	11064 (79.6)
Other	258 (0.9)	133 (0.9)	125 (0.9)
Weight, mean kg (SD)	85.69 (21.79)	85.68 (21.93)	85.70 (21.64)
Body-mass index, kg/m^2			
Mean (SD)	29.29 (6.74)	29.31 (6.82)	29.27 (6.67)
>= 30, n (%)	10657 (38.2)	5406 (38.5)	5251 (38.0)
Risk of COVID-19 Acquisition			
Healthcare Worker or First Responder, n (%)	7605 (27.1)	3833 (27.1)	3772 (27.1)

Table S3: Data from a hypothetical 3 arm trial with 100 cases of COVID-19 on placebo, 20 on mAb and 5 on vaccine. The potential outcomes Y(0),Y(1),Y(2) for the three arms are not directly observable. However, under a monotonicity assumption we can infer the total number that should fall within each of the 3 categories defined by Y(0),Y(1),Y(2).

Y(0),Y(1),Y(2)	# Cases Placebo Arm	# Cases mAb Arm	# Cases Vaccine Arm	Assignment Under Monotonicity	Label
1, 0, 0		?	?	80	Protected by mAb
1, 1, 0	100	20		15	Protected by B/T cells
1, 1, 1			5	5	Doomed
