# Supplementary material for: Examining protective effects of SARS-CoV-2 neutralizing antibodies after vaccination or monoclonal antibody administration
Source: Nat Commun. 2023 Jun 17;14:3605. doi: 10.1038/s41467-023-39292-w (PMC10276829; doi:10.1038/s41467-023-39292-w)
Supplement: Supplementary file 3 — Reporting Summary [file 41467_2023_39292_MOESM3_ESM.pdf]

nature portfolio

Corresponding author(s): Dean Follmann

Last updated by author(s): 24 May 2023

# Reporting Summary

Nature Portfolio wishes to improve the reproducibility of the work that we publish. This form provides structure for consistency and transparency in reporting. For further information on Nature Portfolio policies, see our Editorial Policies and the Editorial Policy Checklist.

## Statistics

For all statistical analyses, confirm that the following items are present in the figure legend, table legend, main text, or Methods section.

| n/a | Confirmed | |
|---|---|---|
| ☐ | ☒ | The exact sample size (*n*) for each experimental group/condition, given as a discrete number and unit of measurement |
| ☒ | ☐ | A statement on whether measurements were taken from distinct samples or whether the same sample was measured repeatedly |
| ☐ | ☒ | The statistical test(s) used AND whether they are one- or two-sided<br>*Only common tests should be described solely by name; describe more complex techniques in the Methods section.* |
| ☐ | ☒ | A description of all covariates tested |
| ☐ | ☒ | A description of any assumptions or corrections, such as tests of normality and adjustment for multiple comparisons |
| ☐ | ☒ | A full description of the statistical parameters including central tendency (e.g. means) or other basic estimates (e.g. regression coefficient) AND variation (e.g. standard deviation) or associated estimates of uncertainty (e.g. confidence intervals) |
| ☐ | ☒ | For null hypothesis testing, the test statistic (e.g. *F*, *t*, *r*) with confidence intervals, effect sizes, degrees of freedom and *P* value noted<br>*Give P values as exact values whenever suitable.* |
| ☐ | ☒ | For Bayesian analysis, information on the choice of priors and Markov chain Monte Carlo settings |
| ☒ | ☐ | For hierarchical and complex designs, identification of the appropriate level for tests and full reporting of outcomes |
| ☒ | ☐ | Estimates of effect sizes (e.g. Cohen's *d*, Pearson's *r*), indicating how they were calculated |

*Our web collection on statistics for biologists contains articles on many of the points above.*

## Software and code

Policy information about availability of computer code

| | |
|---|---|
| Data collection | Medidata Rave was used for COVE and COV-2069 |
| Data analysis | Analyses were performed in R 4.1.0 and 4.2.0. Code at https://github.com/follmand/COVID-19-preventative-efficacy-correlated-with-neuts |

For manuscripts utilizing custom algorithms or software that are central to the research but not yet described in published literature, software must be made available to editors and reviewers. We strongly encourage code deposition in a community repository (e.g. GitHub). See the Nature Portfolio guidelines for submitting code & software for further information.

## Data

Policy information about availability of data

All manuscripts must include a data availability statement. This statement should provide the following information, where applicable:

- Accession codes, unique identifiers, or web links for publicly available datasets
- A description of any restrictions on data availability
- For clinical datasets or third party data, please ensure that the statement adheres to our policy

For COV-2069
Qualified researchers can request access to study documents (including the clinical study report, study protocol with any amendments, blank case report form, and statistical analysis plan) that support the methods and findings reported in this manuscript. Individual anonymised participant data will be considered for sharing once the product and indication has been approved by major health authorities (e.g., US Food and Drug Administration, European Medicines Agency,

Pharmaceuticals and Medical Devices Agency, and so on), if there is legal authority to share the data and there is not a reasonable likelihood of participant re-identification. Requests should be submitted to https://vivli.org/. Regeneron does not commit to a specific timeframe to respond to requests. Requests are vetted in the order that they are received. As for the question regarding restrictions, all data requestors must execute Vivli's Data Use Agreement https://vivli.org/wp-content/uploads/2022/06/2022_06_21-Vivli-Data-Use-Agreement-v1.3.pdf before access is granted. The agreement is publicly available at the link provided. Additionally, the requirement to sign the contractual agreement is noted in REGN's publicly available data sharing policy which can be found here: https://www.regeneron.com/downloads/clinical-trial-disclosure-data-transparency-policy.pdf#:~:text=Regeneron%20is%20committed%20to%20sharing%20clinical%20trial%20data,a%20Regeneron%20sponsored%20study%20by%20submitting%20a%20research

For COVE
As the trial is ongoing, access to participant-level data and supporting clinical documents with qualified external researchers may be available upon request and is subject to review once the trial is complete. Such requests can be made to Moderna Inc., 200 Technology Square, Cambridge, MA 02139, USA. A materials transfer and/or data access agreement with the sponsor will be required for accessing of shared data. All other relevant data are presented in the paper. The protocol is available in the Supplementary Information: Clintrials.gov. NCT04470427. Data requests should have a response within two weeks.

## Research involving human participants, their data, or biological material

Policy information about studies with human participants or human data. See also policy information about sex, gender (identity/presentation), and sexual orientation and race, ethnicity and racism.

| | |
|---|---|
| Reporting on sex and gender | No analyses of sex or gender analyses were conducted. |
| Reporting on race, ethnicity, or other socially relevant groupings | No analyses by race, ethnicity, or other such groups were conducted. |
| Population characteristics | Characteristics are provided in Tables S1 and S2. |
| Recruitment | For COV-2069 Asymptomatic, healthy adolescents (12 to 17 years of age) and adults (≥18 years of age) who were household contacts of an index patient (defined as the first person with a diagnosis of SARS-CoV-2 infection in the household) were eligible to participate if they anticipated living with the index patient for at least 28 days. Participants were recruited via advertisements (flyers, social media, posters) set up by sites/recruitment vendors. For recruitment vendors a prescreener questionnaire was subsequently used prior to connecting subjects with a study site near their location to move into screening.<br><br>For COVE: Eligible participants were persons 18 years of age or older with no known history of SARS-CoV-2 infection and with locations or circumstances that put them at an appreciable risk of SARSCoV-2 infection, a high risk of severe Covid-19, or both. Patients were recruited by flyers, social media, and volunteer registries.<br><br>These are randomized trials so self-selection bias does not apply. |
| Ethics oversight | For COVE the central institutional review board, Advarra, approved the protocol and consent forms. For COV-2069 the central (WCG-IRB) or local IRB of ethics committee at each study center oversaw trial conduct and documentation. |

Note that full information on the approval of the study protocol must also be provided in the manuscript.

# Field-specific reporting

Please select the one below that is the best fit for your research. If you are not sure, read the appropriate sections before making your selection.

☒ Life sciences ☐ Behavioural & social sciences ☐ Ecological, evolutionary & environmental sciences

For a reference copy of the document with all sections, see nature.com/documents/nr-reporting-summary-flat.pdf

# Life sciences study design

All studies must disclose on these points even when the disclosure is negative.

| | |
|---|---|
| Sample size | Sample sizes were 1630 for COV-2069 and 28281 for COVE. COVE was an event driven trial and 151 events provided 90% power to detect a true VE of 60% versus a null hypothesis of 30% VE. The sample size of 30,000 was chosen rapidly achieve 151 events.<br>For COV-2069 a sample size of 1700 was selected to provide 90% power to detect a relative risk of 0.50.<br><br>For the research of this paper, no formal sample size calculations were done as we had to use the data from the finished trials. |
| Data exclusions | No data were excluded from the analysis sets as described in Tables S1 and S2 |
| Replication | No replication of experiments was performed. The data were analyzed using different statistical models to assess consistency of results. These included different functional forms of the PE(Ab) and VE(Ab) models and demonstrated robustness of the conclusions that 1) both VE and PE did correlate with neutralization titer 2) that at lower nAb titer, VE was higher than PE. |

| | |
|---|---|
| Randomization | Participants were randomized to either placebo arm or the prevention arm. |
| Blinding | Both trials were blinded. |

# Reporting for specific materials, systems and methods

We require information from authors about some types of materials, experimental systems and methods used in many studies. Here, indicate whether each material, system or method listed is relevant to your study. If you are not sure if a list item applies to your research, read the appropriate section before selecting a response.

## Materials & experimental systems

| n/a | Involved in the study |
|---|---|
| ☐ | ☒ Antibodies |
| ☒ | ☐ Eukaryotic cell lines |
| ☒ | ☐ Palaeontology and archaeology |
| ☒ | ☐ Animals and other organisms |
| ☐ | ☒ Clinical data |
| ☒ | ☐ Dual use research of concern |
| ☒ | ☐ Plants |

## Methods

| n/a | Involved in the study |
|---|---|
| ☒ | ☐ ChIP-seq |
| ☒ | ☐ Flow cytometry |
| ☒ | ☐ MRI-based neuroimaging |

## Antibodies

| | |
|---|---|
| Antibodies used | Casirivimab and imdevimab (CAS+IMD) is a combination of two neutralising monoclonal antibodies, administered together, that bind non-overlapping epitopes of the SARS-CoV-2 spike protein receptor binding domain. |
| Validation | Casirivimab and imdevimab (CAS+IMD) is a licensed medicine, details are given in https://www.rxlist.com/regen-cov-drug.html<br>See also https://www.science.org/doi/10.1126/science.abd0827 |

## Clinical data

Policy information about clinical studies

All manuscripts should comply with the ICMJE guidelines for publication of clinical research and a completed CONSORT checklist must be included with all submissions.

| | |
|---|---|
| Clinical trial registration | COVE ClinicalTrials.gov number, NCT04470427. COV-2069 ClinicalTrials.gov, NCT04452318. |
| Study protocol | The protocol for COVE is available in the supplementary materials of Baden, Lindsey R., et al. "Efficacy and safety of the mRNA-1273 SARS-CoV-2 vaccine." New England journal of medicine 384.5 (2021): 403-416. For COV-2069, the protocol is in the supplementary materials of O'Brien, Meagan P., et al. "Subcutaneous REGEN-COV antibody combination to prevent Covid-19." New England Journal of Medicine 385.13 (2021): 1184-1195. |
| Data collection | COVE was conducted at 99 sites in the United States. Participants received their first injection between 27 July and 23 October 2020.<br><br>COV-2069 was conducted at 112 sites in the United States, Romania and Moldova. The study was initiated on 13 July 2020 and completed on 4 October 2021 |
| Outcomes | This research used the primary outcome of the COVE and COV-2069 trial; symptomatic COVID-19. Infection and asymptomatic infection for COV-2069 was a secondary outcome. They outcomes were defined in the protocols (see above) The primary outcome for each trial is provided in the supplementary materials. |
